# Attentional biases, as measured by motion-induced blindness, are linked to schizophrenia traits

**DOI:** 10.1371/journal.pone.0325609

**Published:** 2025-06-24

**Authors:** Joshua Paton, Jeroen J. A. van Boxtel

**Affiliations:** 1 Discipline of Psychology, School of Health Sciences, Faculty of Health, University of Canberra, Bruce, ACT, Australia; 2 Turner Institute for Brain and Mental Health, School of Psychological Sciences, Monash University, Clayton, Victoria, Australia; Federal University of Paraiba, BRAZIL

## Abstract

Typically, people demonstrate a small attentional bias towards the left visual field. This bias has not consistently been observed in schizophrenia. Schizophrenia has been thought to be linked to a top visual field bias, due to an impaired dorsal stream found in those individuals. Here we assessed left/right and upper/lower spatial biases measuring perceptual disappearances in a motion-induced blindness (MIB) task and link those to schizophrenia traits. The sample were consisted of first year psychology students (*N *= 54; 22 males, 31 females, and 1 prefer not to say; age 18–54 years; median age = 23). Schizophrenia traits were measured using the schizotypal personality questionnaire (SPQ) and perceptual aberration scale (PAS). We found that higher SPQ scores correlated with a top field bias. Higher interpersonal scores (an SPQ subscore) linked to a right field, and so did PAS (*p* = .007). Higher cognitive-perceptual scores linked to a left field bias. Taken together, this study supports a complex relation between spatial attention and schizophrenia traits in MIB, in which a top field bias may reflect an impaired dorsal stream. A possible implication of these findings is that MIB may serve as a potential tool for screening early schizophrenia traits.

## 1. Introduction

The assessment of schizophrenia emphasizes the importance of presentation, duration, and severity of symptoms when considering a diagnosis [1]. Many assessment tools have been created to assist in diagnosing, including the Wisconsin schizotypy scales, schizotypal personality questionnaire (SPQ), and positive and negative syndrome scale [1–3]. However, onset of the condition can make it difficult to diagnose schizophrenia, due to its emergence in late adolescence, a period where depression, anxiety, and social withdrawal can be frequent [4]. This overlap of symptoms can increase the chance of misdiagnosis. Consequently, it is advantageous to develop additional measures such as those that utilize perceptual/cognitive tasks to help diagnose schizophrenia. Below we will review spatial attention and biases, and dorsal stream functioning in schizophrenia, and identify MIB as a potentially useful measure in this context. We aim to identify the future possibility of using motion-induced blindness (MIB) as a supplemental tool to current approaches in the context of investigating schizophrenia traits.

Rather than investigating a clinical group versus a control group, we focus on traits along a continuum to examine the existing degree of perceptual abnormalities related to schizophrenia in the general population. Screening tools such as the SPQ and the perceptual aberration scale (PAS) have traditionally been implemented to measure traits in individuals who are at increased risk of diagnosis [5–16]. Therefore, using a non-clinical sample would be most appropriate. Indeed, these measures have been used extensively in non-clinical samples previously, including the original study for the construction of the SPQ [16], multiple studies involving the SPQ brief revised and updated versions [6,11,12], and a 10-year longitudinal study that used the PAS in measuring psychotic-like symptoms [7].

Schizophrenia is a mental health condition characterized by positive and negative symptoms, disorganized thinking and speech, and disorganized/abnormal motor behaviour. Important among these symptoms are abnormalities in perception (i.e., hallucinations and delusions) and deficits in attention (i.e., tangentiality, dissociation, and agitation) [1]. In terms of perception, hallucinations are commonly reported in schizophrenia, and they are superficially similar to visual illusions. However, while both are a form of mistaken perception, visual illusions are a healthy response of the brain to some stimuli [17–20]. A real-life example helps to explain MIB in everyday life. As you are driving along a highway or country road, after a long period of time you may find yourself fixated on what is in front of you, and you become completely unaware of your surroundings; this is similar to MIB, and has been a line of investigation in driving scenarios [21]. Research suggests for visual illusions to be effective, integrity of the visual system is required [18]. Therefore, those with an impaired visual system would fail to experience illusory effects to the same level a healthy individual would. Notredame et al. [18] highlighted the use of visual illusions to explore normal and impaired perception. They likened hallucinatory symptoms of schizophrenia to visual illusions, with the difference that they are not initiated by a stimulus, but instead are a by-product of the condition itself [18]. Visual illusions may therefore allow insight into the mechanisms of schizophrenia.

Resistance to visual illusions may also be related to problems with attention [20,22,23]. For example, attentional capture leads to our attention being drawn to specific stimuli in our environment. Those with schizophrenia have been reported to demonstrate heightened attentional capture: an inability to effectively ignore irrelevant information [20,22–25]. In one study, patients with and without schizophrenia were asked to locate a target shape in their visual field, while ignoring a distractor that moved randomly or changed colour. The results showed the patient group was more distracted by the distractor than the control group, suggesting an impaired ability to inhibit reflexive shifts of attention amongst the schizophrenia group [24]. Therefore, individuals with schizophrenia may become distracted by peripheral stimuli and fail to fixate on what is in front of them. This distraction may translate to resistance to the visual phenomena of MIB, as the task requires directed attention (usually towards a fixed point in the visual field) [17,26–35]. If such results could be replicated, the possible implications could suggest that those with schizophrenia have more trouble processing motion in spatial attention.

These attentional and motion processing problems could be related to the dorsal visual stream. The dorsal stream has long been proposed to be crucial for attention, and visual processing of motion and location [36–44]. It is often called the ‘where’ pathway in reference to these functions [33,39,45]. Indeed, a dorsal stream deficiency has long been thought to be linked to schizophrenia [23–25,38,39,41,43–47], especially in relation to motion and location processing [41,43,45–47]. Studies (particularly functional MRI) have supported the primary role of the dorsal stream in motion processing [33,39,43–45,47,48].

Attentional issues relevant for the current report, are related to ‘pseudoneglect’. Pseudoneglect is a commonly observed attentional bias to the left visual field in the typically developing population [32,36,37,42,49–57]. Pseudoneglect makes reference to the medical condition hemispatial neglect, which is characterized by a complete neglect of the left field caused by brain lesions found in the parietal lobe of the right hemisphere [42]. Researchers have shown pseudoneglect is related to the right hemisphere in the brain [36,42,51,53,54,56,58–61]. The role of the right side of the brain is for the allocation of attention [51,56]. As the brain works contralaterally [59], a left field bias reflects the typical dominance of the right hemisphere [36,42,51,53,54,56,58–61]. Conversely, an impaired right hemisphere of the brain could present via a reduced or absent pseudoneglect. Indeed, pseudoneglect is less commonly observed in those clinically diagnosed with schizophrenia [36,42,49,53,54]. Supporting this idea further, brain imaging has found abnormalities in the right hemisphere of the schizophrenia brain and decreased pseudoneglect [20,22,23,36,37,39,42,44,47,49,53,57].

Apart from horizontal (left/right) visual biases, the literature also highlights the existence of vertical (top and bottom visual field) biases. Early studies on focal brain lesions revealed the dorsal stream allocates attention to the bottom visual field, while the ventral stream allocates attention to the top visual field [55,62]. Indeed, dorsal parts of early visual cortex predominantly represents bottom visual field [19,62,63]. It has been suggested that the dorsal pathway (important for (inter)action with the outside world [64]) attends more to stimuli in the bottom visual field, due to their generally closer proximity and manipulable location. Meanwhile, the ventral (what) pathway attends to upper spatial stimuli due to the need for the stimuli to be located/identified from a distance [56,57]. Although there is evidence for a bias of attention to the bottom field in the healthy population [39–41,43,44], there is currently no research on how this bias is affected in schizophrenia. Given the literature-suggested deficit in dorsal processing, reduced attention to the lower visual field is predicted to relate to schizophenia [23–25,38,39,41,43–47].

**Fig 1:**
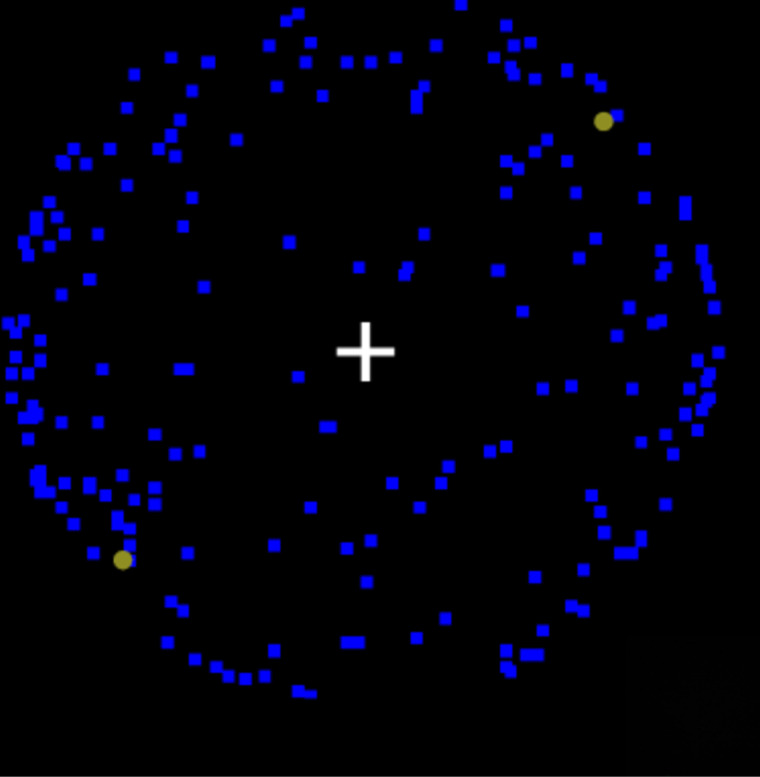
Motion-induced Blindness Paradigm. Yellow Dots Disappear when Fixated on the White Cross.

Given the reviewed findings, MIB may contribute to existing approaches to identify perceptual atypicalities in schizophrenia. This is so, because MIB is a visual phenomenon in which salient stimuli spontaneously disappear and reappear from visual awareness when superimposed on a moving background of distractors [17,19,27] (Fig 1). Further, MIB is readily observed by healthy typically developing observers [17–20]. Directed attention results in increased disappearances of peripheral stimuli (i.e., yellow dots) [17,26–35], and MIB has been used to research spatial biases in attention (i.e., pseudoneglect) [17,21,27,49,50,56,57]. MIB is therefore a motion-based stimulus influenced by (spatial) attentional biases, and presumably relies heavily on dorsal stream processing, thus combining possible atypical cognitive and perceptual functions in schizophrenia. MIB could, therefore, allow one to obtain additional insights into processing differences in schizophrenia.

Previous research has investigated relationships between MIB perception and schizophrenia. That research has shown that positive symptoms (i.e., hallucinations and delusions) enhanced MIB [20], while negative symptoms (i.e., depression) and cognitive disorganization tended to reduce MIB [20]. Other research has shown overall less MIB disappearances amongst individuals with schizophrenia [65]. However, to date, there have been no investigations into the horizontal and vertical spatial biases in schizophrenia using MIB. Possibly this is due to the previous layouts used in MIB not being ideal to study a thorough quantification of visual field biases [32,57]. This is because studies mostly used a traditional triangular formation of disappearing targets [17]. They mostly included two dots in the top field, and one central dot below. However, a triangular model is not ideal for investigating spatial biases, as lower left and right quadrants are not investigated. Few studies have used a square formation [21,32] even though it allows for deeper analysis into the interactions between the horizontal and vertical fields. Therefore, we employ the square formation in this study.

Here, we investigate the relationship between schizophrenia traits in a non-clinical population and perception (measured by the number of MIB disappearances), as well as spatial attention (measured by the location in which the disappearances occur). We measure schizophrenia traits using SPQ and PAS. We focused our hypotheses and analyses on SPQ and not PAS (on which we performed exploratory analyses), because of two reasons. Firstly, the SPQ was specifically created based on the DSM-III-R criteria for schizophrenia traits, which are still relevant for contemporary diagnosis [15,66,67]. Secondly, the PAS is a measure for general psychotic-like symptoms [9]. This property of the PAS includes other disorders that have psychotic-like symptoms (i.e., bipolar), and is thus less specific to schizophrenia spectrum disorders.

Based on the literature we expected 1) higher SPQ scores to result in fewer MIB disappearances, because of the literature-suggested impairment in the dorsal stream that is found amongst individuals with schizophrenia traits; 2) a significant effect of SPQ on the left field vs right field (horizontal) biases, in which less of a left field bias would be observed as SPQ scores increased; 3) there would be a significant effect of SPQ on the top field vs bottom field (vertical) biases, in which a top field bias would be evident amongst individuals with higher SPQ scores.

## 2. Method

### 2.1. Participants

Participants were recruited via convenience sampling. First year psychology students were recruited and awarded course credit for participation. The participants had to have self-reported normal or corrected-to-normal vision. No other inclusion and exclusion criteria were employed. The sample was made up of 54 participants (22 males, 31 females, and 1 participant preferred not to say). Age ranged from 18 to 54, *M* = 23, *SD* = 7.38. No missing data was recorded. The recruitment period begun on the 13^th^ of May 2022 and ended on the 11^th^ of August 2022. Informed (written) consent was obtained from all participants prior to the beginning of the online study.

As there is no agreed-upon sample size estimation for generalized linear mixed models (GLMM) [68], we used a sample size estimation for a repeated measures ANOVA to decide the required power, using G*Power [69]. Since GLMMs are considered more powerful than ANOVA’s [70], our estimate was a conservative one. Firstly, we used a partial eta-squared of 0.06 from the McEwen et al. [32] study, which used a similar design, investigating the interaction between ADHD group and horizontal and vertical biases in MIB disappearances. We calculated partial eta-squared from the raw data from that study (https://osf.io/m8zxc/files/osfstorage), when performing a mixed ANOVA with the factors: ADHD group (high/low; median split on Conners Adult ADHD Rating Scales: < 52 low, ≥ 52 high) and location (same 4 locations as in the current study). Critical alpha was set at 0.05, and a desired power at 0.80. The calculations estimated a required sample size of 34. We exceeded this with our sample of 54 participants. This increase sample size was obtained because we had set out a data collection window, and finished earlier collecting the sample size that the power analysis indicated. We decided to continue collecting, so that we could potentially find less strong effects.

### 2.2. Materials

#### 2.2.1 Schizotypal personality questionnaire-brief revised (updated).

For our study, we implemented the SPQ-BRU to measure participants’ schizophrenia traits. This revision had small changes in wording that improved psychometrics of the test in terms of increasing factor loadings, and reducing residual variance, when compared with earlier versions of the questionnaire [12]. The SPQ asks participants to score each question on a 5-point scale (strongly disagree – strongly agree), in which higher scores indicate higher schizotypy. The questionnaire covers several domains associated with schizophrenia, in which the SPQ has three factors made up of nine sub-scales in total: interpersonal factors (no close friends, constricted affect, and social anxiety), cognitive perceptual factors (ideas of reference, suspiciousness, magical thinking, and unusual perceptions), and disorganized factors (eccentric behaviour, and odd speech).

The SPQ has demonstrated excellent psychometrics. Its internal consistency conveys excellent reliability, in which coefficients range from .87 to .94 for SPQ factor scores, while also ranging from .83 to .93 for SPQ subscale scores [6]. Additionally, two-month test-retest reliabilities range from .86 to .95, once again demonstrating excellent psychometric properties [16]. Meanwhile, Callaway et al. [6] were able to demonstrate sound construct validity amongst most SPQ domains including cognitive-perceptual factors, no close friends, constricted affect, and social anxiety. The SPQ is originally based off the DSM-III [16]. However, it is still a relevant contemporary measure, because the current DSM outlines similar criteria to the older versions (such as hallucinations, delusions, and incoherence).

SPQ scores in our sample (*M* = 90.43, *SD* = 20.94) were comparable to normative data (*M* = 81.64, *SD* = 17.79) [71], though perhaps slightly higher. Recent work by Glisker [71] in a similar age-group as ours showed similar descriptives (*M* = 91.85, *SD* = 18.59).

#### 2.2.2 Perceptual aberration scale (PAS).

The PAS measures participants’ body-image and perceptual aberration traits. The PAS was chosen because of its ability to measure perceptual features of schizophrenia such as out-of-body experiences, alterations of size and shape of their bodies, and the feeling one’s organs are rotting [8]. The scale consists of 35-items that cover five key areas that are uncommon in the general population: 1) unclear boundaries of the body, 2) feelings of unreality or estrangement of parts of one’s body, 3) feelings of deterioration of one’s body, 4) perceptions of change in the size, relative proportions, or spatial relationships of one’s body parts, and 5) changes in the appearance of the body [8]. Participants were asked to answer either true or false (one or zero points, respectively). Studies by Chapman et al. [7] have found test-retest reliability to be between.75 to.85. Other studies have also shown sound validity including concurrent, discriminant, predictive, and criterion [9,13,72].

PAS scores in our sample (*M *= 9.19, *SD *= 6.29) were lower than normative data (*M *= 15.60, *SD* = 6.92) [72]. This difference may suggest a sampling bias in our data, and could be cause of a floor effect. The discrepancies between our data and normative data suggest that the SPQ is probably a more reliable measure compared to the PAS. While these were not reasons to use the SPQ over the PAS (as we made that decision a priori), they do support the use of PAS for exploratory analyses.

### 2.3. Stimulus

The experiment was conducted online, using ‘Gorilla Experiment Builder’ (www.gorilla.sc). Descriptions below describe how the animation was constructed, but for example, stimulus size would have depended on the participants’ set-up. However, to compensate for any potential variability that may have been introduced depending on the participants’ set-up, it was encouraged that participants sat approximately 30 centimetres from the screen. Additionally, the experiment was limited to only laptops and desktops; therefore, ruling out use of smaller screens such as phones or tablets, that may have added more variability.

The MIB visual trials included all possible 15 different combinations of where yellow dots appeared (i.e., upper left [UL], upper right [UR], lower right [LR], and lower left [LL]), allowing for trials with 1,2, 3, or 4 yellow dots. Stimuli were presented on a black background. The moving stimulus included 192 blue squares (5x5 pixels; 0.1 cm x 0.1 cm) rotating as if placed with a uniform random distribution on a virtual sphere (8 cm x 7.5 cm). Yellow dots (10-pixel diameter; 0.2 cm x 0.2 cm) were equally spaced across each quadrant and were placed just within visual projection of the sphere. Blue and yellow are conventional colours [17] and work well within the paradigm. Brightness of the yellow dots (opacity) were set through pilot testing, to ensure about half of the dots disappeared during a trial. This was to avoid floor and ceiling effects as much as possible. A static white cross (0.7 cm x 0.7 cm) in the centre of the screen served as a fixation point for participants. Fig 1 demonstrates an example frame of an MIB stimulus with two targets. The experiment was run in full-screen mode, with the stimulus taking up 100% of the screen.

### 2.4. Procedure

The ethics application for this study was approved by the University of Canberra Human Research Ethics Committee-11597. Students who chose to enrol in the study were taken to the external site ‘Gorilla Experiment Builder’ (www.gorilla.sc), where the experiment was hosted [73]. From here, participants were provided with general information outlining the project, confidentiality, data storage, risks, and consent. Once participation was agreed upon by the participant, they were asked demographic questions such as gender and age. Next, instructions were provided to explain how the MIB trials were to be completed. Participants were instructed to fixate on the white cross. Each trial began once the spacebar was pressed. There were a total of 200 randomized trials, each lasting four seconds. Once a trial finished, participants were asked to select the yellow dots that were visible at the end of each trial by clicking on small markers on a response screen at the four possible locations of the yellow dots (i.e., UL, UR, LL, LR). Furthermore, to prevent fatigue and possible eye strain, participants were encouraged to take self-paced breaks in between trials when necessary. Participants completed eight practice trials to become familiar with the process before beginning the recorded trials. After the MIB task, participants completed the SPQ and the PAS. To limit any response bias (i.e., social desirability) in the questionnaires, their true nature was disguised, and renamed to the ‘perception of self’ questionnaire and the ‘body sensation’ questionnaire, respectively.

### 2.5. Data analysis

Because participants indicated which locations they perceived a dot, we could calculate which dots had perceptually disappeared. For example, the dots that were physically present, but not reported as visible. We also checked that participants did not have too many “false alarms” (i.e., reporting a dot as visible where no dot was presented). Several incorrect responses would suggest the individual did not sincerely respond, and this action would be more evident towards the end of the trials where fatigue/boredom may have set in. The second half of participants’ responses were checked to ensure there were no more than 3 incorrect responses for dots that were not presented in the preceding trial. No participants were excluded based on this criterion.

Participants were excluded if they had completed the entire experiment in under 15 minutes, as it was calculated that the minimum time required for the MIB trials was almost 13 minutes. Three participants were excluded based on this criterion. The reported number of participants in this study is that after these exclusions were implemented.

### 2.6. Statistical analysis

The data was analysed using the program jamovi version 2.3.2 [74]. We performed a generalized linear mixed model (GLMM) using the GAMLj module. We used a binomial distribution and logit link function (i.e., a logistic regression), which is most appropriate for our binary dependent variable ‘disappearance’ of a dot (0 visible, 1 invisible). Two independent variables for locations were created, for which we coded the locations of the dots (UL, UR, LL, LR) into Horizonal (left/right), and Vertical (top/bottom). Another, continuous independent variable was SPQ (or PAS in other analyses). The model included a random intercept to account for individual differences (coded as the identifier, ID, of a participant). The final model was: Disappearances ~ 1 + Vertical + Horizontal + SPQ + Vertical:Horizontal + Vertical:SPQ + Horizontal:SPQ + Vertical:Horizontal:SPQ + (1 | ID), where colons indicated interactions between terms.

The GLMM therefore analysed the effect of schizophrenia traits (measured by the SPQ and PAS) and target location (lower/upper/left/right) on the strength of the MIB illusion (i.e., the disappearance of a dot). The GLMMs included both horizontal and vertical factors, but are written up in separate sections for ease of discussion.

## 3. Results

### 3.1. SPQ as a main effect against disappearances

**Fig 2:**
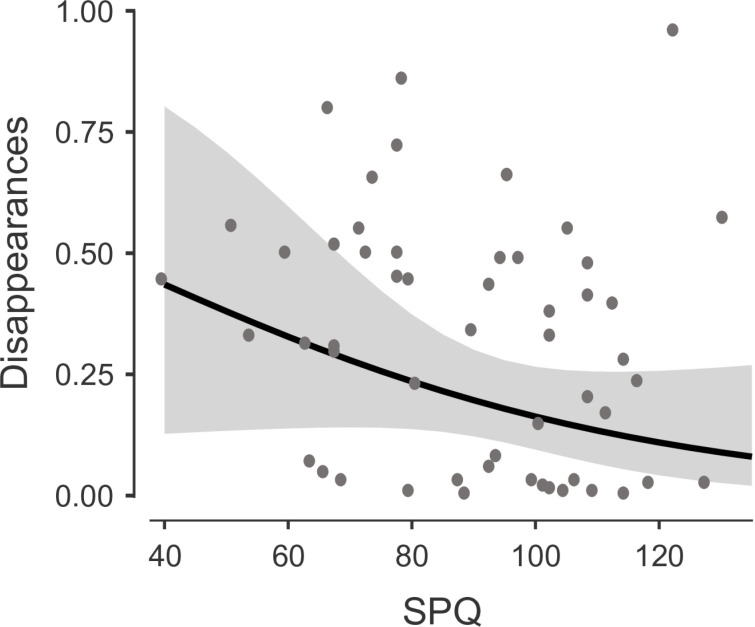
Scatter Plot of SPQ Against Average of MIB Disappearances. The grey dots represent participant averages.

We tested whether SPQ predicted MIB disappearances. Firstly, beta estimates (β = −0.023 [CI = −0.053 to 0.007], SE = 0.016) and a visual inspection of a scatterplot (Fig 2) suggested higher SPQ scores resulted in less perceptual disappearances. However, counter to our hypothesis, the GLMM revealed that SPQ scores were not a significant predictor for MIB disappearances (χ^2^(1) = 2.18, *p* = .139).

### 3.2. The interaction between SPQ scores and horizontal locations against disappearances

Our second hypothesis predicted that an interaction between SPQ scores and horizontal locations, in which higher SPQ scores would demonstrate a decreased left field bias (less pseudoneglect). This hypothesis was not supported by the data. The GLMM indicated that the interaction between SPQ and Horizontal location was not significant (χ^2^(1) = 0.49, *p* = .485). Therefore, less pseudoneglect amongst higher SPQ scores could not be concluded.

### 3.3. The interaction between SPQ scores and vertical locations against disappearances

**Fig 3:**
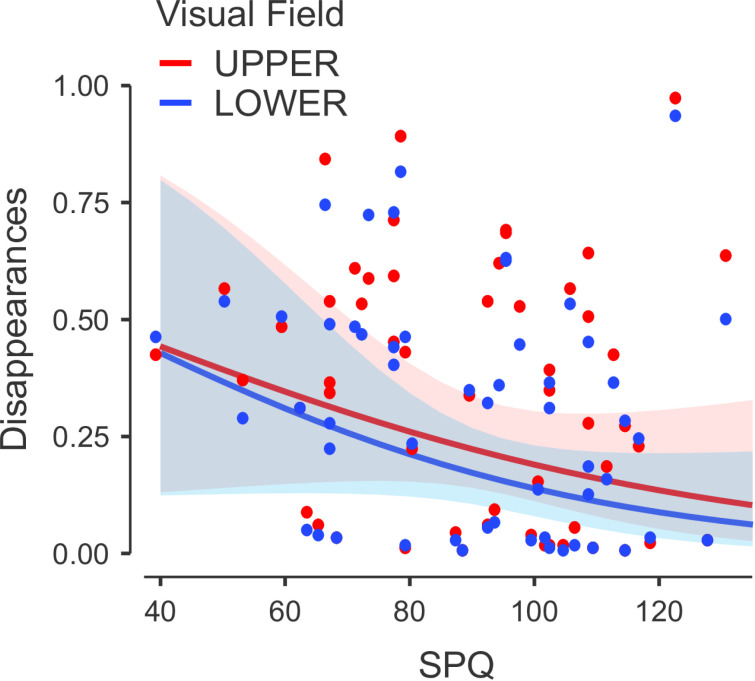
Scatter Plot of SPQ and Top Field vs Bottom Field Against Average of MIB Disappearances. A blue and red dot pair represents each participant.

Our final hypothesis stated an interaction between SPQ scores and vertical locations would be a predictor for MIB disappearances, in which higher SPQ scores would be correlated with a top field bias. The interaction was significant (χ^2^(1) = 12.61, *p* < .001). Hence, the number of MIB disappearances was dependent on this interaction (β = −0.005 [CI = −0.008 to 0.002], SE = 0.001). Additionally, a visual inspection of the data implied a top field bias amongst higher scoring participants (Fig 3). This evidence together, supported our third hypothesis.

## 4. Exploratory analyses

### 4.1 PAS scores

A GLMM was run for PAS scores against MIB disappearances with horizontal and vertical locations included. The results of the GLMM showed a significant interaction between PAS and vertical locations (χ^2^(1) = 4.48, *p* = .034), demonstrating a similar top field bias as the SPQ interaction. Additionally, a significant interaction between PAS and horizontal locations (χ^2^(1) = 7.31, *p* = .007) was observed, which was not found in the SPQ analyses. Those individuals with lower PAS scores demonstrated a left field bias on average, but as PAS scores increased, a right field bias emerged.

### 4.2 SPQ factors

The SPQ and PAS are suggested in the literature to be correlated [75], and this was so also in our data (*r *= .65, *p *< .001). To understand why the SPQ and PAS show different effects in our data, SPQ factors (cognitive-perceptual, interpersonal, and disorganized [16]) were explored separately. A correlation matrix revealed that cognitive-perceptual (*r *= .64), interpersonal (*r *= .60), and disorganized (*r *= .61) shared a significant moderate relationship with the PAS.

**Fig 4:**
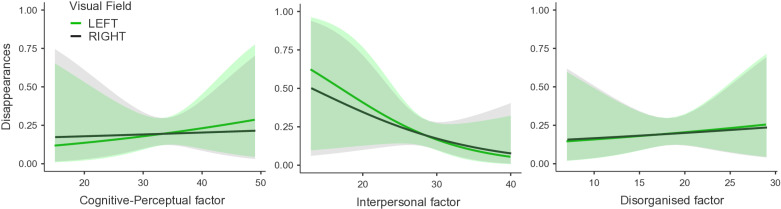
Effects Plot. Cognitive-Perceptual Factor (left), Interpersonal Factor (middle) and Disorganized Factor (right), and Left Field vs Right Field Against MIB Disappearances. The shaded area refers to 95% confidence intervals of the two lines (group means).

A GLMM for each factor demonstrated cognitive-perceptual (χ^2^(1) = 9.86, *p* = .002) and interpersonal (χ^2^(1) = 11.36, *p* < .001) factors had significant interactions with horizontal locations. Interestingly, cognitive-perceptual demonstrated a negative beta estimate (β = −0.024 [CI = −0.040 to –0.009, SE = 0.008), while interpersonal had a positive beta estimate (β = 0.032 [CI = 0.013 to 0.050], SE = 0.009). These results imply that the cognitive-perceptual factor had a stronger right field bias at lower scores, and a stronger left field bias at higher scores (Fig 4). On the contrary, the interpersonal factor had a stronger left field bias at lower scores, and a stronger right field bias at higher scores (Fig 4). Meanwhile, the disorganized factor was not significant (χ^2^(1) = 0.73, *p* = .394; Fig 4).

To conclude, although SPQ had a moderate significant relationship with its factors and the PAS, SPQ overall did not show a significant interaction with horizontal location. Although, two of its factors (cognitive-perceptual and interpersonal) did, but in opposite directions.

## 5. Discussion

Our study investigated the influence of schizophrenia traits on spatial biases in perceptual disappearances (and attention) in a MIB paradigm.

### 5.1. SPQ and its factors

Although studies including the SPQ and MIB are limited, there is a moderate amount of literature supporting weaker MIB in schizophrenia [20,35,65]. Although a significant effect did not exist in our data, the trend was in the same direction, with higher SPQ scores resulting in weaker MIB. Perhaps, because we researched schizophrenia traits, and not a sample of individuals diagnosed with schizophrenia, our effect is less strong.

We investigated the interaction between traits and the vertical spatial biases in disappearances, and therefore attention. Our results showed that as SPQ scores increased, so did a bias towards the top field. Since a bias to the bottom visual field is associated with intact dorsal stream function, our results are consistent with previous literature that hypothesizes that schizophrenia is associated with deficits in dorsal stream [39–41,43,44].

We also predicted higher traits would show less pseudoneglect (left field bias) on MIB trials, based on literature suggesting a right hemispheric dysfunction in schizophrenia [36,42,51,53,54,56]. However, our results did not reflect a significant effect. These results do not replicate literature findings, in which the majority of studies were able to demonstrate limited pseudoneglect in schizophrenia.

This unusual finding warranted further exploration. We therefore looked at the three factors of the SPQ: cognitive-perceptual, interpersonal, and disorganized [15]. Our rationale was that possibly one or more of the factors showed opposite effects on disappearance. Significant links between both cognitive-perceptual and interpersonal factors and SPQ were found, but not with the disorganized factor. Interestingly, the cognitive-perceptual factor showed that higher scores were associated with an increase left field bias. This goes against the reduced left field bias that is generally observed in schizophrenia [36,42,53,54,56]. However, the link to the cognitive-perceptual factor was not investigated in those studies. There does not appear to be existing literature to explain the positive correlation between the cognitive-perceptual factor and left field bias, which is therefore an interesting topic for future study. The interaction between the interpersonal factor and horizontal locations was significant, and suggested that there was less pseudoneglect as scores increased. This was consistent with what we expected to see between overall SPQ scores and horizontal locations. Though speculative at the moment, the correlation between interpersonal traits and pseudoneglect may exist because the right hemisphere has been found to be important for social interaction (i.e., recognizing faces [52]). Our significant results that show less pseudoneglect when considering the interpersonal factor, could be an indirect reflection of right hemisphere dysfunction in schizophrenia. If such a finding could be rigorously replicated, it could suggest that the interpersonal factor may help identify social deficits and right hemispheric dysfunction in schizophrenia; while also being a good measure of spatial biases in general. This last point is supported by Chen et al. [10] who found that the interpersonal factor is associated with poorer attention, while the cognitive-perceptual factor was not. Our results highlight that considering the cognitive-perceptual, interpersonal, and disorganized factors, in addition to the overall SPQ score, could be beneficial in perception studies.

### 5.2. PAS

Unlike the SPQ, the PAS had significant interactions with both horizontal and vertical locations. This difference was surprising because SPQ and PAS share a moderate correlation as per our study and the literature [75]. Therefore, it was speculated the PAS would show similar results to the SPQ analysis. Our speculative interpretation of these findings is the PAS and the interpersonal factor of the SPQ share similar characteristics of self-perception and how others perceive oneself, because the origins of the PAS include a strong emphasis on perception of body-image [8]. Indeed, the PAS and interpersonal factor share a strong significant correlation in our data.

### 5.3. Spatial biases

Aside from our focus on schizophrenia, our results also complement studies that have demonstrated differences in attention measured by MIB. For instance, our analysis confirmed that there are differences in MIB disappearances between the top and bottom visual field. The exploratory analyses highlighted that perceptual differences between the left and right visual field also exist. These findings are in line with increased perceptual disappearances in different patterns in spatial attention [21,27,32]. From implications in driving and distraction [21,57], our ability to attend to stimuli [27], possible links to mental disorders such as schizophrenia and ADHD [32], and even explanations for medical conditions such as hemispatial neglect [42]; MIB has proven that it can be empirically and practically reliable in measuring spatial biases. Our findings demonstrate the possible usefulness of MIB illusions as a tool to measure mechanisms and concepts including spatial biases, and atypical perception and attention. Based on our results, MIB perceptual disappearances, and specifically vertical biases, have potential to be good measures for atypical perception, and be possibly reflective of positive symptoms and dorsal stream dysfunction in schizophrenia. Our findings also raise some speculative possibilities. For example, horizontal field biases could be measures for deficits in interpersonal and social traits, as reflected in the interactions between the PAS and the SPQ interpersonal factor. This finding may reflect negative symptoms found in schizophrenia, such as social anxiety, inability to recognize faces and emotions, and difficulty socializing [1].

A possible limitation of our study is that we had a non-clinical sample, using schizophrenia-like traits measures. This may limit our ability to generalize to the schizophrenia population. However, using traits is also an advantage. This is because any of the effects are likely to be smaller, and thus our findings are likely to be stronger in a schizophrenia cohort. Relatedly, in our study we correlated MIB to SPQ scores, with SPQ score being used as a proxy for schizophrenia. The additional value of MIB over SPQ in schizophrenia diagnosis would still need to be thoroughly established in samples with schizophrenia diagnosis. Additionally, future directions should also consider measures including Symptoms of Schizophrenia Inventory [76] and Symptom Self-rating Scale for Schizophrenia [77].

The nature of the online setting of our experiment may too be a limitation, and most likely increases the chances of variability in the data and decreases the chances of finding significant effects. However, several post-pandemic studies have shown little to no differences in data replicability between in-lab and online experiments [78–84], and indeed our findings align well with the current literature. Furthermore, we increased the sample number to help combat the potentially lower-quality online data.
